# Caught in the Whirl: Computed Tomography Diagnosis of Transverse Colon Volvulus With Proximal Jejunal Involvement and Cecocolic Pneumatosis

**DOI:** 10.7759/cureus.110219

**Published:** 2026-06-03

**Authors:** El Mahdi Choukri, Zakaria Boulahcen, Youssef Kacimi, Amine Naggar, Siham Alaoui Rachidi

**Affiliations:** 1 Diagnostic and Interventional Radiology, Mohammed VI University Hospital, Tangier, MAR; 2 Radiology, Faculty of Medicine and Pharmacy, Abdelmalek Essaâdi University, Tangier, MAR

**Keywords:** acute abdomen, bird-beak sign, bowel obstruction, cecocolic pneumatosis, computed tomography, mesenteric whirl sign, transverse colon volvulus

## Abstract

Transverse colon volvulus is a rare cause of mechanical bowel obstruction, and preoperative diagnosis may be challenging because clinical and radiographic findings are often nonspecific. Computed tomography (CT) plays a central role in identifying the transition point, mesenteric whirl sign, associated bowel involvement, and signs of bowel compromise.

We report the case of a 65-year-old man with chronic obstructive pulmonary disease who presented with a five-day history of bowel obstruction, including absence of stool and flatus and vomiting. Upright abdominal radiography showed colonic air-fluid levels, prompting further CT evaluation. Abdominopelvic CT demonstrated marked dilatation of the small bowel and right colon upstream from a left paramedian mesenteric whirl. The volvulus involved the transverse colon, which showed a bird-beak configuration, and an adjacent proximal jejunal loop. The superior mesenteric vein was displaced to the left of the superior mesenteric artery at the level of the twist. Cecocolic pneumatosis was present, suggesting bowel compromise, without pneumoperitoneum, portal venous gas, mesenteric venous gas, or definite mural nonenhancement.

Emergency surgery was performed for transverse mesenterico-colic volvulus, and the operative procedure documented ileocecal resection with stoma creation. The patient later underwent ileostomy takedown with manual end-to-end ileocolic anastomosis. This case highlights the value of CT in diagnosing rare volvulus patterns, defining the involved bowel segments, and identifying complications that support urgent surgical management.

## Introduction

Transverse colon volvulus is an uncommon cause of mechanical bowel obstruction. Compared with sigmoid and cecal volvulus, it is less frequently encountered and may be difficult to diagnose preoperatively because the clinical presentation is often nonspecific and may overlap with other causes of acute bowel obstruction [1-5]. Delayed recognition can be clinically significant, as volvulus may progress to closed-loop obstruction, bowel ischemia, perforation, and peritonitis.

Plain abdominal radiography may suggest obstruction by showing bowel dilatation and air-fluid levels, but it is often insufficient to identify the precise transition point or the mechanism of obstruction. Computed tomography (CT) is therefore essential because it can demonstrate the mesenteric whirl sign, bird-beak configuration, involved bowel segments, and associated complications such as pneumatosis intestinalis, free fluid, pneumoperitoneum, or portomesenteric venous gas [6-11].

In this context, the whirl sign refers to twisting of bowel loops, mesentery, and mesenteric vessels around a torsion point; the bird-beak sign describes abrupt tapering of the obstructed bowel loop; closed-loop obstruction refers to obstruction of a bowel segment at two points; and portomesenteric venous gas refers to gas within the portal venous or mesenteric venous system, which may indicate advanced bowel compromise when associated with concerning clinical and CT findings.

We report a case of transverse mesentericocolic volvulus involving the transverse colon and a proximal jejunal loop, complicated by cecocolic pneumatosis. The aim of this case is to highlight the radiologic value of CT in recognizing an unusual volvulus pattern, assessing signs of bowel compromise, and guiding urgent surgical management.

## Case presentation

A 65-year-old man with a four-year history of chronic obstructive pulmonary disease, currently off treatment, was admitted to the emergency surgical unit with symptoms of bowel obstruction. Symptoms had evolved over five days and included absence of stool and flatus associated with vomiting, without fever, and with clinical deterioration.

On admission, clinical examination showed signs of dehydration. The patient was eupneic, with oxygen saturation of 96% on room air, and bilateral wheezing was noted on chest auscultation. Hemodynamically, the heart rate was 102 beats/min and blood pressure was 100/80 mmHg, without signs of shock. Neurological examination was unremarkable. Initial laboratory tests showed hemoconcentration, with hemoglobin of 18.2 g/dL and hematocrit of 57.2%, leukocytosis of 14,700/mm³ with neutrophilia, and elevated C-reactive protein at 186 mg/L. The elevated hemoglobin and hematocrit were interpreted in the acute setting as hemoconcentration related to clinical dehydration. Given the history of chronic obstructive pulmonary disease, chronic secondary polycythemia was also considered; however, it could not be established in the absence of baseline hemoglobin values or documented chronic hypoxemia.

Initial upright abdominal radiography demonstrated marked right colonic distension associated with dilated small-bowel loops. Several central abdominal air-fluid levels were noted, with an additional right colonic air-fluid level. No free subdiaphragmatic air was identified. These findings were consistent with bowel obstruction and prompted further evaluation with abdominopelvic CT (Figure 1).

**Figure 1:**
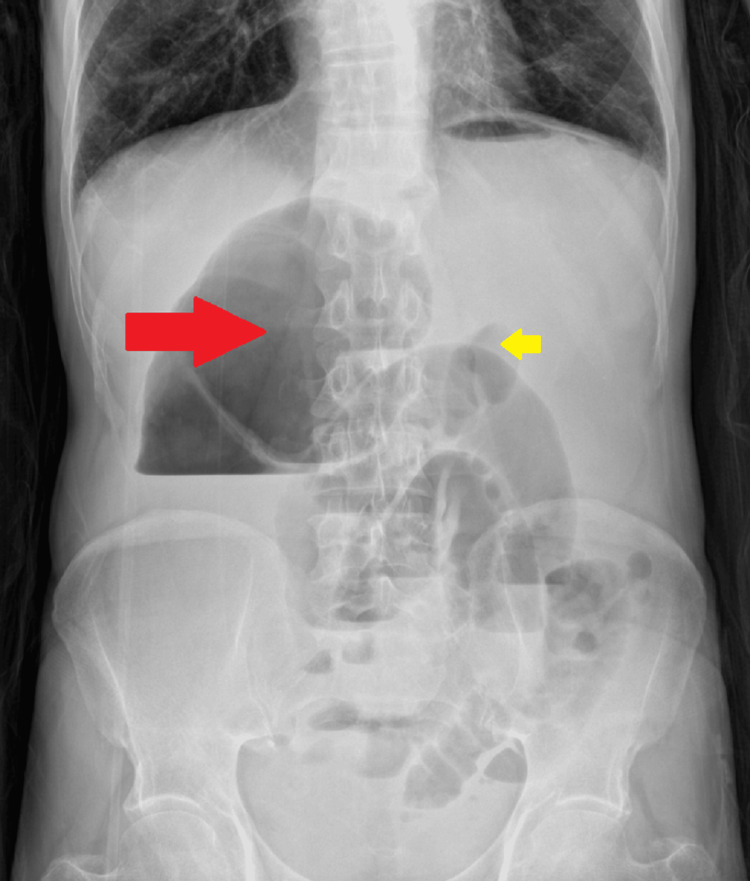
Upright abdominal radiograph showing mixed small-bowel and right-sided colonic obstruction Upright abdominal radiograph demonstrates marked right colonic distension with a prominent right-sided air-fluid level (red arrow), associated with dilated small-bowel loops (yellow arrow) and additional central abdominal air-fluid levels. No free subdiaphragmatic air is identified.

Abdominopelvic CT was performed before and after intravenous contrast administration, with multiplanar reformations. The unenhanced acquisition was mildly degraded by respiratory motion artifact, without substantial impairment of diagnostic interpretation. Noncontrast CT images demonstrated marked distension of the right colon and adjacent small-bowel loops, with a prominent air-fluid level within the dilated cecocolic segment. Coronal reformations confirmed marked cecocolic distension, measuring up to approximately 81.4 mm in maximal diameter (Figure 2).

**Figure 2:**
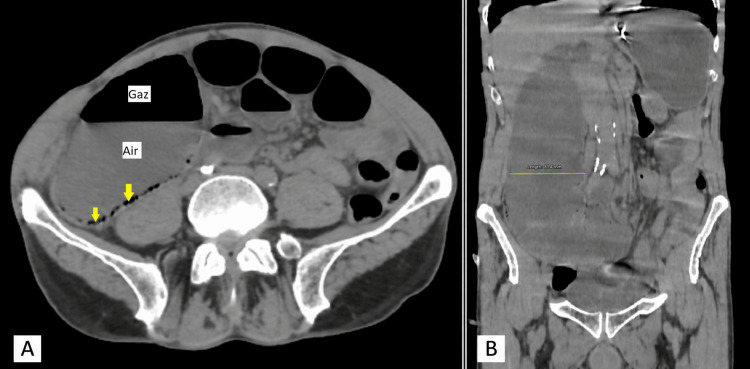
Unenhanced CT showing marked cecocolic distension with an air-fluid level and cecocolic pneumatosis Unenhanced CT images are mildly degraded by respiratory motion artifact without substantial impairment of diagnostic interpretation. (A) Axial image demonstrates marked cecocolic distension with a prominent air-fluid level; yellow arrows indicate intramural gas within the cecal and right colonic wall, consistent with cecocolic pneumatosis. (B) Coronal reformation confirms marked cecocolic distension, measuring up to approximately 81.4 mm in maximal diameter. CT, computed tomography

Portal venous phase CT further characterized the obstruction by demonstrating a left paramedian mesenteric whirl sign centered on the twisted mesenteric pedicle. At the level of torsion, there was abrupt tapering of the involved transverse colonic segment with caliber disparity between the dilated upstream bowel loop and the decompressed downstream bowel loop. The transverse colon was involved in the torsion, with associated involvement of an adjacent proximal jejunal loop. Signs of bowel compromise were present, including intramural gas within the cecal and right colonic wall, consistent with cecocolic pneumatosis. A small amount of intraperitoneal fluid was present in the right iliac fossa, with mild mesenteric fat stranding. No pneumoperitoneum, portal venous gas, mesenteric venous gas, or definite mural hypoenhancement was identified (Figure 3).

**Figure 3:**
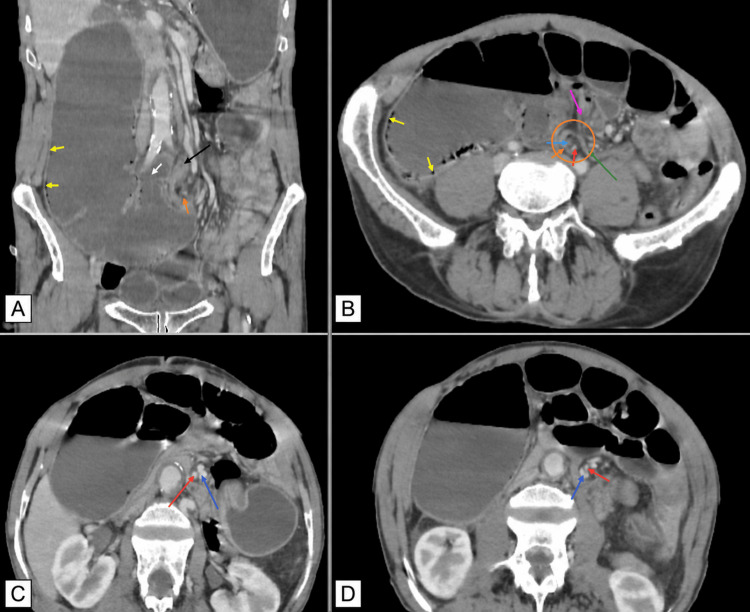
Portal venous phase CT showing transverse mesenterico-colic volvulus with bowel caliber disparity, a mesenteric whirl sign, vascular involvement, and cecocolic pneumatosis Portal venous phase contrast-enhanced CT further characterizes the volvulus and its complications. (A) Coronal reformation demonstrates marked cecocolic distension with intramural gas in the cecal and right colonic wall, consistent with cecocolic pneumatosis (yellow arrows). The ileocecal valve is identified (white arrow). A left paramedian mesenteric whirl sign is seen (orange arrow), associated with an abruptly narrowed downstream colonic segment (black arrow), illustrating bowel caliber disparity across the torsion point. (B) Axial image demonstrates cecocolic pneumatosis (yellow arrows), the corresponding mesenteric artery (red arrow), the corresponding mesenteric vein (blue arrow), and the mesenteric whirl sign (orange circle). A dilated upstream bowel loop proximal to the torsion point is indicated by the pink arrow, while a decompressed downstream bowel loop distal to the torsion point is indicated by the green arrow. (C, D) Axial images focus on the twisted mesenteric vascular pedicle, showing the mesenteric artery (red arrows) and mesenteric vein (blue arrows) involved in the torsion. No pneumoperitoneum, portal venous gas, mesenteric venous gas, or definite mural hypoenhancement is identified. CT, computed tomography

Oblique axial and coronal maximum-intensity-projection CT reformations focused on the mesenteric vascular pedicle demonstrated twisting of the implicated mesenteric arterial and venous structures at the level of the volvulus, supporting the vascular component of the whirl sign (Figure 4).

**Figure 4:**
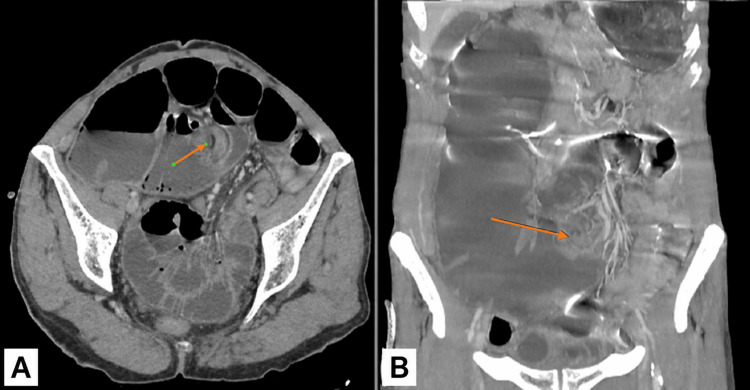
Oblique axial and coronal MIP CT reformations showing twisting of the mesenteric vasculature within the volvulus Portal venous phase oblique MIP CT reformations focus on the mesenteric vascular pedicle involved in the torsion. (A) Oblique axial MIP image demonstrates twisting of the mesenteric vascular pedicle at the level of the volvulus (orange arrow). (B) Oblique coronal MIP image confirms the spiral configuration of the mesenteric vascular pedicle within the volvulus (orange arrow), highlighting the vascular component of the whirl sign. CT, computed tomography; MIP, maximum intensity projection

These CT findings supported the diagnosis of mechanical bowel obstruction secondary to transverse mesentericocolic volvulus involving the transverse colon and a proximal jejunal loop, with cecocolic pneumatosis suggesting bowel compromise.

The patient subsequently underwent emergency surgery on 20/04/2025. Intraoperative assessment confirmed transverse mesentericocolic volvulus. The operative procedure documented ileocecal resection with stoma creation, followed by transfer to the visceral surgery department. No additional operative detail was available regarding the separate resection or preservation of the transverse colon. Intraoperative photographs demonstrated the volvulated bowel segment and the resected specimen, providing direct surgical correlation with the preoperative CT findings (Figure 5). Histopathological details were not documented in the available medical file.

**Figure 5:**
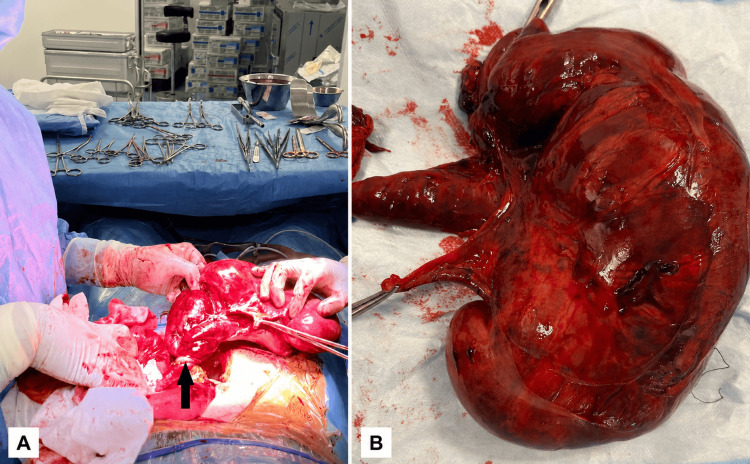
Intraoperative correlation of transverse mesenterico-colic volvulus with the resected congested bowel (A) Intraoperative photograph demonstrates the volvulated bowel segment (black arrow), providing direct surgical correlation with the preoperative CT diagnosis of transverse mesenterico-colic volvulus. (B) Gross specimen photograph after resection shows a markedly distended and congested bowel segment, corresponding to the operative decision to perform ileocecal resection with stoma creation. CT, computed tomography

During a later admission for restoration of bowel continuity, the patient underwent ileostomy takedown, adhesiolysis of ileoparietal and coloparietal adhesions, section of the colonic and small-bowel ends, manual end-to-end ileocolic anastomosis, and drainage near the anastomosis in the right iliac fossa.

The available follow-up documentation indicates that restoration of bowel continuity was performed after the initial emergency surgery, reflecting clinical stabilization after the acute episode. The latter operative course included ileostomy takedown and manual end-to-end ileocolic anastomosis, with drainage near the anastomosis in the right iliac fossa. No postoperative follow-up CT, detailed discharge summary, or histopathological report was documented in the available medical file.

## Discussion

Transverse colon volvulus is an uncommon cause of mechanical bowel obstruction and remains less frequently encountered than sigmoid or cecal volvulus [1-5]. Because its clinical presentation is often nonspecific, diagnosis may be delayed, especially when initial imaging demonstrates only bowel dilatation and air-fluid levels. This represents a clinical limitation of the case, as the presenting symptoms were nonspecific and primarily suggested acute bowel obstruction; the specific diagnosis depended on CT identification of the transition point, whirl sign, involved bowel segments, and associated complications. CT was decisive in demonstrating a left paramedian mesenteric whirl, a bird-beak configuration of the transverse colon, associated proximal jejunal involvement, and cecocolic pneumatosis.

The whirl sign is one of the most important CT findings in volvulus. It reflects twisting of bowel loops, mesentery, and mesenteric vessels around a torsion point [6-9]. In this patient, the whirl involved the transverse colon and an adjacent proximal jejunal loop, explaining the combined colonic and small-bowel dilatation. Compared with most reported transverse colon volvulus cases, which mainly present as large-bowel obstruction, the associated proximal jejunal involvement explains the mixed small-bowel and colonic obstruction pattern observed in this case [1-5]. A plausible mechanism is traction or incorporation of an adjacent jejunal loop into the torsion complex at the mesenteric whirl, rather than an isolated colonic twist. This interpretation remains radiological, as the available operative documentation confirmed transverse mesenterico-colic volvulus but did not separately describe the jejunal mesentery. This mixed pattern is important because it may mimic other causes of acute obstruction, including internal hernia, closed-loop small-bowel obstruction, or midgut volvulus. The abnormal position of the superior mesenteric vein to the left of the superior mesenteric artery supported mesenteric twisting in this acute setting. However, this finding should be interpreted cautiously and should not be used alone to diagnose congenital malrotation without additional anatomic confirmation.

The presence of cecocolic pneumatosis was the major CT sign suggesting bowel compromise. The measured cecocolic distension contributed to the assessment of obstruction severity, particularly in the context of volvulus; however, concern for bowel compromise was based on the combined presence of marked upstream distension, cecocolic pneumatosis, mild free fluid, mesenteric fat stranding, and inflammatory laboratory abnormalities rather than bowel diameter alone. Pneumatosis intestinalis can occur in both benign and life-threatening conditions, and its significance depends on the clinical and imaging context [10,11]. In this case, pneumatosis was associated with mechanical obstruction, volvulus, mild free fluid, and mesenteric fat stranding, raising significant concern for bowel compromise. The absence of pneumoperitoneum was reassuring against established perforation, while the absence of portal venous gas, mesenteric venous gas, and definite mural non-enhancement argued against advanced transmural bowel infarction on CT; however, these findings did not exclude clinically relevant bowel compromise. Therefore, the CT findings supported urgent surgical management. Leukocytosis with neutrophilia and markedly elevated C-reactive protein reinforced concern for an inflammatory or ischemic complication in the clinical context of obstruction and CT-proven volvulus, although these laboratory abnormalities were nonspecific and could not independently establish bowel ischemia.

Management of transverse colon volvulus is generally surgical, particularly when there are signs of bowel compromise, peritonitis, perforation, or failed decompression [1-6]. Unlike sigmoid volvulus, for which endoscopic decompression may be considered in selected stable patients without signs of ischemia, transverse colon volvulus is rare, and management is less standardized. In this patient, the combination of volvulus, marked upstream dilatation, and cecocolic pneumatosis supported urgent surgical management. The available operative documentation recorded ileocecal resection with stoma creation, followed later by ileostomy takedown and manual end-to-end ileocolic anastomosis.

This case has limitations. Histopathological details and detailed postoperative follow-up imaging or a discharge summary were not documented in the available medical file. Therefore, direct histopathological correlation of CT signs of bowel compromise and detailed assessment of postoperative evolution were not possible.

This case highlights three radiological teaching points. First, mixed small-bowel and right colonic dilatation on abdominal radiography should prompt CT evaluation to identify the transition point and mechanism of obstruction. Second, careful analysis of the whirl sign is essential to determine which bowel segments are involved. Third, pneumatosis in the setting of volvulus should be considered a warning sign, even in the absence of pneumoperitoneum or portomesenteric venous gas. CT, therefore, plays a central role not only in diagnosing rare volvulus patterns but also in assessing complications that influence surgical decision-making.

## Conclusions

Transverse mesenterico-colic volvulus is an uncommon cause of mechanical bowel obstruction that may be difficult to diagnose based on clinical and plain radiographic findings alone. CT is essential for identifying the mesenteric whirl sign, the bird-beak transition, the involved bowel segments, and associated signs of bowel compromise.

In this case, CT demonstrated a left paramedian whirl involving the transverse colon and a proximal jejunal loop, with upstream cecocolic and small-bowel dilatation and cecocolic pneumatosis. These findings supported urgent surgical management, with the available operative documentation recording ileocecal resection with stoma creation. This case emphasizes the value of CT in recognizing rare volvulus patterns and providing key information for timely surgical decision-making.
